# Dissecting the Complexities of Glucose Signaling in Yeast

**DOI:** 10.1371/journal.pbio.0020151

**Published:** 2004-05-11

## Abstract

xx

An organism's survival depends on developing effective strategies for identifying and adapting to available sources of food. Even organisms as small as the budding yeast Saccharomyces cerevisiae can respond in a complex way to the presence of different energy sources. While yeast can metabolize many different sugars, glucose provides the highest energy yield. To achieve this energy efficiency, yeast cells rely on specialized enzymes and metabolic states that mediate glucose metabolism by sensing and responding to this sugar. The presence of glucose triggers a rapid and dramatic change in the expression of about a quarter of S. cerevisiae's 5,500 genes.[Fig pbio-0020151-g001]

**Figure pbio-0020151-g001:**
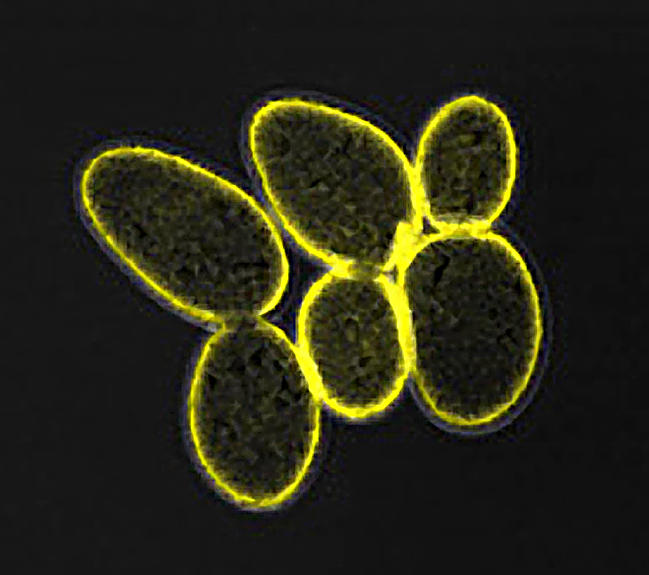
Ras at the plasma membrane of S. cerevisiae

Many of the cellular participants in the glucose “sense-and-response” pathway have been identified, but their exact relationships remain unknown. The gene expression response to glucose has been previously characterized through the use of a cDNA microarray, which allows simultaneous assessment of the transcriptional state of every gene. In research reported in this issue, James Broach and his colleagues at Princeton University have attempted to connect the individual components in this complex pathway by performing microarray analysis on a series of mutants (yeast strains with defects in specific proteins). They link certain portions of the response to known proteins and begin to understand how the pieces of this pathway fit together.

The researchers initially focused on two proteins, called Ras2 and Gpa2. These proteins have been previously implicated in the transcriptional response to glucose and are members of a well-established family of signaling proteins. To investigate the roles of these proteins in the response, the researchers used mutants of Ras2 and Gpa2 that could be activated on demand and then performed microarray analysis to see which genes responded to activation of these proteins. They found that even in the absence of glucose, activation of either protein induced a transcriptional profile almost identical to the profile generated in yeast exposed to glucose. This shows that the complex and dramatic transcriptional response to glucose can be recapitulated by the activation of a single protein.

The Ras2/Gpa2 pathway, however, is not the whole story. The group then went on to show that not all glucose-responsive genes are regulated in the same manner. They found that another pathway, independent of Ras2 and Gpa2, is able to elicit a portion of the transcriptional response to glucose. The partial redundancy of these pathways is a curious phenomenon and bears further investigation.

The authors have achieved an initial step in mapping the topology of the intricate signaling pathway (or pathways) involved in the response to glucose. Furthermore, the approach—that of using microarray analysis of mutants in a pathway to deduce the mechanisms of regulation—will be useful in efforts to map other complex responses in yeast as well as in higher organisms.

